# Molecular Assembly between Weak Crosslinking Cyclodextrin Polymer and *trans*-Cinnamaldehyde for Corrosion Inhibition towards Mild Steel in 3.5% NaCl Solution: Experimental and Theoretical Studies

**DOI:** 10.3390/polym11040635

**Published:** 2019-04-08

**Authors:** Yucong Ma, Baomin Fan, Tingting Zhou, Hua Hao, Biao Yang, Hui Sun

**Affiliations:** 1 School of Materials and Mechanical Engineering, Beijing Technology and Business University, Beijing 100048, China; mayucong18@163.com (Y.M.); jzfbm@163.com (T.Z.); sunhui@th.btbu.edu.cn (H.S.); 2 Institute of Chemistry, Chinese Academy of Sciences, Beijing 100190, China; haohua@iccas.ac.cn

**Keywords:** β-cyclodextrin, weak crosslinking polymer, *trans*-cinnamaldehyde, corrosion inhibitor, molecular dynamics simulation, quantum chemistry

## Abstract

Constructing molecular assembly between a soluble cyclodextrin polymer (SCDP) and an anticorrosive component is conducive to increasing the availability of a corrosion inhibitor with low molecular polarity in aqueous solution. The SCDP was prepared via the weak crosslinking effect of glutaraldehyde using β-cyclodextrin as the subunit, whose structure was confirmed by proton nuclear magnetic resonance spectra (^1^H NMR), X-ray diffraction and morphology. An assembly between SCDP (host) and *trans*-cinnamaldehyde (guest, CA) was constructed, and the intermolecular interactions were disclosed by Fourier transform infrared spectra (FTIR). The corrosion inhibition of SCDP/CA assembly for mild steel in 3.5% NaCl solution was assessed through electrochemical and surface analyses. ^1^H NMR results showed that exterior hydroxyls of β-cyclodextrin were the active sites for crosslinking. Hydrogen bonds might be the binding force between SCDP and CA according to FTIR analyses. Electrochemical measurements revealed that SCDP/CA assembly could suppress both cathodic and anodic reactions and enhance the polarization impedance for mild steel in the corrosive medium with a maximum efficiency of 92.2% at 30 °C. Surface analyses showed that CA molecules could be released from the assembly followed by the energy competition mechanism, and solely adsorb on the steel surface in parallel form, which was further evidenced by theoretical modeling.

## 1. Introduction

Cyclodextrins (CDs) comprise a family of macrocyclic oligosaccharides manufactured from starch, in which several glucose units are connected by α-1,4-glycosidic bonds in a cyclic manner [1,2]. The architecture of CDs is similar to a truncated cone with a hydrophobic cavity and hydrophilic periphery, which has many attractive functions such as hydrolysis, catalysis, molecular recognition and polymerization [3]. The commonly used CDs are α-, β- and γ-CD possessing six, seven and eight glucose units, respectively. Among all the CDs, β-CD is frequently utilized as a host compound because of its proper molecular dimensions (height of 0.79 ± 0.01 nm, exterior diameter of 1.54 ± 0.04 nm) that are suitable for a raft of guest moieties to form a supramolecular complex [4]. The host–guest complex can prevent the labile xenobiotics from oxidation and enhancing their chemical stability in ambient environments. In recent years, crosslinking polymers based on various CDs has attracted a great deal of attention and relevant studies on their ability to construct supramolecular assembly through molecular recognition and self-assembly have been performed [5]. Much success had been achieved in the application of cyclodextrin polymers in the insoluble resin or gel form. For instance, Tang et al. [6] prepared two insoluble cyclodextrin polymers based on β-CD and γ-CD, respectively, applying tetrafluoroterephthalonitrile as the crosslinking agent, which could be used as the effective adsorbents for rapidly removing the endocrine disrupting chemicals in water. Xiao et al. [7] developed a cyclodextrin polymer and uncovered the selective determination of a trace amount of diclofenac in water by its displacement of fluorescent dye encapsulated in the polymer. Mizuno and co-workers [8] synthesized a monochloro triazine modified β-CD crosslinking polymer with three dimensional interconnected porous structures by the phase separation technique, and utilized it as the filler in the flow-through column to remove typical environmental pollutants. Compared with the extensive research on CD-based polymers in an insoluble resin or gel form, very limited studies have been carried out on the preparation and functionalization of soluble cyclodextrin crosslinking polymers (SCDP).

Mild steel is widely used in various industrial and engineering applications due to its excellent structural and mechanical properties. However, corrosion of mild steel in practical conditions, especially in a chlorine-containing environment, is a serious challenge, which can cause prominent damage to the metal and degrade its properties, thereby limiting its applications [9]. The inhibition of mild steel in corrosive media occurs through the following strategies: anticorrosive coating, surface treatment, cathodic/anodic protection and using corrosion inhibitors [10]. These approaches have the capability of mitigating corrosion of mild steel in diversified corrosive media, among which, using corrosion inhibitors is one of the most efficient methods of preventing metals from corrosion. Organic corrosion inhibitors have the advantages of high efficiency, diverse functions and being environmentally benign, as compared to the inorganic counterparts such as phosphate and nitrate [11]. In general, organic inhibitors inhibit the corrosion of metals by interacting with surface atoms via adsorption through the lone pair electrons of heteroatoms (O, N, S and P), the π-orbitals in a conjugated system, electron density and the electronic structure of the molecule [12,13]. *trans*-Cinnamaldehyde (CA) is one of the α-, β-unsaturated aldehydes, which can be extracted from cassia bark [14]. Cabello et al. [15] evaluated the corrosion inhibition effect of CA allied with carbon monoxide for high–nickel and –iron alloys in HCl solution at high pressure and temperature, and pointed out that a thin polymerized film could be formed on the metal surface to retard the corrosion under an extreme environment. Avdeev and co-workers [16] employed a series of unsaturated aldehydes as the inhibitors for corrosion retardation of mild steel in HCl and H_2_SO_4_ media. They found that CA chemically adsorbed on the steel surface and was the most efficient inhibitor among all the studied aldehydes in a wide range of concentrations for mild steel in hot acid solutions (60–95 °C). However, CA cannot effectively disperse and dissolve in water under normal pressure and temperature owing to its low molecular polarity, and thus, it hardly exhibits a corrosion inhibition effect for metals. 

Supramolecular assembly based on CDs has attracted enormous attention, and detailed works have been devoted to many aspects, such as pharmaceutics [17], environmental protection [18], batteries [19] and polymer science [20]. Whereas, the theory of supramolecular chemistry (molecular recognition and self-assembly) has not been adequately applied in the realm of corrosion inhibition, especially corrosion inhibitors. In our previous studies [21,22,23], several simple host–guest complexes were prepared based on octadecylamine and CDs, which were used as the effective corrosion inhibitors for mild steel in corrosive media. In those studies, we also found the stability of the resulting complexes were unsatisfactory during the application period.

It has been documented that cyclodextrin crosslinking polymers provide the strong stabilization effect for guest molecules [5]. Apart from this, few studies have reported the construction of molecular assembly between SCDP and anticorrosive component coupling with the corrosion inhibition characterizations. Therefore, in this work, β-CD subunits were weakly crosslinked by flexible glutaraldehyde molecules to prepare SCDP, which was featured by proton nuclear magnetic resonance spectra (^1^H NMR), X-ray diffraction (XRD) and morphological observation from scanning electron microscopy (SEM). The molecular assembly was prepared between SCDP and CA through the solution method, with the non-covalent interactions clarified by Fourier transform infrared spectra (FTIR). The corrosion inhibition effect of SCDP/CA assembly for mild steel in 3.5% NaCl solution was collectively evaluated by electrochemical measurements and surface analyses. The raised inhibition mechanism of the assembly derived from experimental results was evidenced by molecular dynamics simulations and quantum chemistry calculations. This work combines areas of polymer science and corrosion inhibition, which might provide the experimental and theoretical experience in constructing supramolecular assemblies based on CDs with certain functions. 

## 2. Materials and Methods

### 2.1. Materials and Solutions

Analytical grade β-CD, CA, glutaraldehyde, and D_2_O were obtained commercially from Innochem Company (Beijing, China). The absolute ethanol, NaCl, and Na_2_CO_3_ provided by Beijing Chemical Works (Beijing, China) were also of analytical purity. All chemicals were used as received without further purification.

The lab-made deionized water with a resistivity of 18.2 MΩ·cm (25 °C) was used to prepare all solutions in this study. As a special case, 3.5% NaCl (mass ratio) solution, employed as the corrosive medium, was prepared by dissolving solid NaCl in the deionized water. Q235A mild steel coupons with a dimension of 5.0 × 2.5 × 0.2 cm^3^ (Tianjin Institute of Chemical Research and Design, Tianjin, China) were used as the metal substrates, the composition of which is listed in Table S1. 

### 2.2. Preparation and Characterization of Soluble Cyclodextrin Crosslinking Polymer

**Scheme 1:**
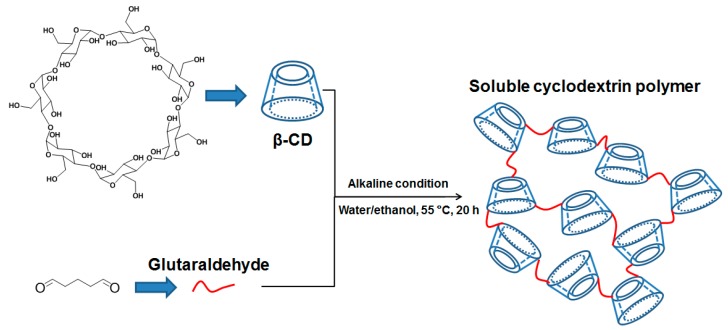
Schematic representation of the weak crosslinking of β-CD to obtained the soluble cyclodextrin polymer.

The soluble cyclodextrin crosslinking polymer (SCDP) was prepared via the non-covalent crosslinking between the carbonyl groups on flexile glutaraldehyde and hydroxyl groups on β-CD in the alkaline environment (Scheme 1). A clean 500 mL three-neck round flask was charged with β-CD, glutaraldehyde and the appropriate amount of deionized water/ethanol (7/1, *v*/*v*) solution, in which the molar ratio of β-CD/glutaraldehyde is 4:1. Vigorous stirring at 40 °C allowed β-CD and glutaraldehyde to become entirely soluble in water/ethanol solution. Subsequently, a controlled amount of 0.5 mol/L Na_2_CO_3_ solution was dribbled into the mixture to adjust the pH value between 11 and 12. Prior to the crosslinking reaction, the mixture in the flask was bubbled by N_2_ for 5 min. Then, the mixture was vigorously stirred under 1000 rpm at 55 °C for 20 h to complete the weak crosslinking polymerization. A reflux unit was connected to the flask in order to prevent the crosslinking agent (glutaraldehyde) from volatilizing. The homothermal procedure at 60 °C for 2 h was conducted to realize the uttermost weak crosslinking of β-CD. The gel resultant was filtered off by the 0.45 μm mixed resin membrane. The filtrate was further dialyzed through a cellulosic membrane (pore size: approximate 5 nm) to remove water, salt by-product and free β-CD, and then freezing dried to obtain the solid SCDP with a yield of 58.6%. 

Appropriately weighed β-CD and SCDP were dissolved in D_2_O and then transferred to an AVANCE III HD style nuclear magnetic resonance spectrometer (400 MHz, Bruker, Karlsruhe, Germany). The chemical shifts of protons for β-CD before and after crosslinking were recorded. XRD patterns for β-CD and SCDP were determined via the X’Pert Powder style X-ray diffractometer (PANalytical, Almelo, The Netherland) operated at a voltage of 30 kV. The samples were analyzed in the 2*θ* range from 5° to 60° with a step size of 0.1°. After being sputtered with a thin gold layer, the morphologies of β-CD and SCDP were examined by a QUANTA FEG 250 style scanning electron microscope (FEI, Hillsboro, OR, USA) at 20 kV accelerating voltage.

### 2.3. Preparation and Characterization of Molecular Assembly

The molecular assembly based on SCDP and CA was constructed following the known solution method [22]. In brief, an excess amount of SCDP was dissolved in deionized water and this was followed by dropping CA-containing ethanol solution (0.2 mol/L) under stirring; the solution was thermostatically shaken at 40 °C for 32 h, let to settle in order to remove free CA, and freeze dried to get the assembly. Subsequently, SCDP/CA assembly was transferred into a pallet with spectroscopic grade KBr powder for FTIR characterization in a Nicolet iN10 MX model spectrophotometer (Thermo Scientific, Waltham, MA, USA) with the wave number of 4000–400 cm^−1^.

In order to qualitatively analyze the stability and thermodynamic parameters of SCDP/CA assembly, the simple host–guest complex between β-CD and CA, as the crosslinking unit of assembly, was prepared by the typical procedure reported in the literature [24]. Phase solubility studies of CA with different concentrations of β-CD in water were performed at pre-specified temperatures according to the method raised by Higuchi and Connors [25]. The optimal configuration of the β-CD/CA complex was also explored by molecular dynamics simulation, which is described in detail in Section 2.6.

### 2.4. Electrochemical Measurements

Prior to the electrochemical tests, the steel specimens were degreased by absolute ethanol; afterwards, SiC papers (up to 2000#) and fine Al_2_O_3_ particles (0.5 μm) were successively applied to abrade and polish the specimens. All electrochemical measurements were conducted through an Autolab PGSTART 302N style electrochemical workstation (Metrohm, Switzerland) in a classical three-electrode cell equipped with a steel specimen of 1 cm^2^ exposure area as the working electrode, a saturated calomel electrode (SCE) as the reference electrode and a platinum sheet as the auxiliary electrode. The mentioned potentials in the context were all versus SCE. 

Before potentiodynamic polarization and electrochemical impedance spectroscopy (EIS) tests, the working electrode was immersed in 3.5% NaCl solution for 45 min to achieve the stabilization of open circuit potential (*E*_ocp_). Cathodic and anodic polarization curves were recorded starting from *E*_ocp_ to −200 and to +250 mV, respectively, at a scan rate of 1 mV/s. EIS measurements were conducted at *E*_ocp_ in a frequency range from 100 kHz to 10 mHz using an amplitude of 10 mV (peak to peak). For checking the stability and linearity of EIS tests, linear polarization resistance (*R*_pl_) was examined by scanning the potential of *E*_ocp_ ± 10 mV with a scan rate of 0.166 mV/s. Triplicate tests were conducted for each experimental level to guarantee the reproducibility. NOVA 2.1 software was employed to analyze the corresponding electrochemical parameters.

### 2.5. Surface Analyses

The steel specimen was incised into pieces of 1.0 × 1.0 × 1.0 cm^3^, which were polished as per the protocol described in Section 2.4 for posterior surface analyses. The small pieces of mild steel were mounted in a RCC-III style rotating corrosion instrument (Qinyou Company, Gaoyou, China) and immersed in 3.5% NaCl solution with and without different concentrations of additives (SCDP or SCDP/CA assembly). The rotating corrosion tests were performed at 30 °C for 72 h with a rotating speed of 70 r/min to simulate the practical flowing condition. The morphologies of samples were captured by SEM with the same experimental conditions as described in Section 2.2. The spectra of attenuated total reflection infrared spectra (ATR-FTIR) for samples, after rotating corrosion tests, were recorded by the infrared instrument aforementioned in Section 2.3 from 4000 to 600 cm^−1^ with a 4 cm^−1^ spectral resolution.

### 2.6. Theoretical Calculations

Theoretical simulations were performed with Materials Studio 8.0 software (BIOVIA Inc. San Diego, CA, USA). Concerning the high calculation cost and low efficiency of SCDP/CA assembly, we chose one crosslinking unit, β-CD/CA simple complex, as the simulating target to qualitatively analyze the energy and structural characteristics of the assembly. The structure of the β-CD molecule was acquired from Cambridge Structural Database (reference code: BCDEXD03); meanwhile, the structure of CA was obtained from PubChem Database. The energies of possible configurations of the β-CD/CA complex were evaluated by the Dynamics tool in COMPASS force field under NVT ensemble (constant atom number, constant volume and constant temperature maintained by Andersen thermostat) at 303 K. The simulation period of energy analyses lasted for 300 ps, which was sufficient for configurations to achieve equilibrium. 

The adsorption mechanism for the β-CD/CA complex on the steel surface was also illuminated by molecular dynamics simulation using the Quench tool in Forcite module at 303 K for 500 ps with a step of 1 fs. Five Fe (1 1 0) layers were cleaved for the adsorption process due to its dense and stable structure [26], which was frozen during simulation. A simulation box with a dimension of 3.4 × 3.4 × 3.7 nm^3^ containing 500 H_2_O molecules and one β-CD/CA complex was built to probe the interaction between the complex and iron atoms.

Quantum chemical calculations based on density functional theory were employed to support the adsorption mechanism of the β-CD/CA complex on Fe (1 1 0) obtained from molecular dynamics simulation. The quantum descriptors of the CA molecule, such as optimal configuration, mapping of molecular electrostatic potential (MEP), highest occupied molecular orbital (HOMO) and lowest unoccupied molecular orbital (LUMO) were calculated in a solvent (water) environment under an energy optimized state.

## 3. Results and Discussions

### 3.1. Characterization of Soluble Cyclodextrin Polymer

**Figure 1:**
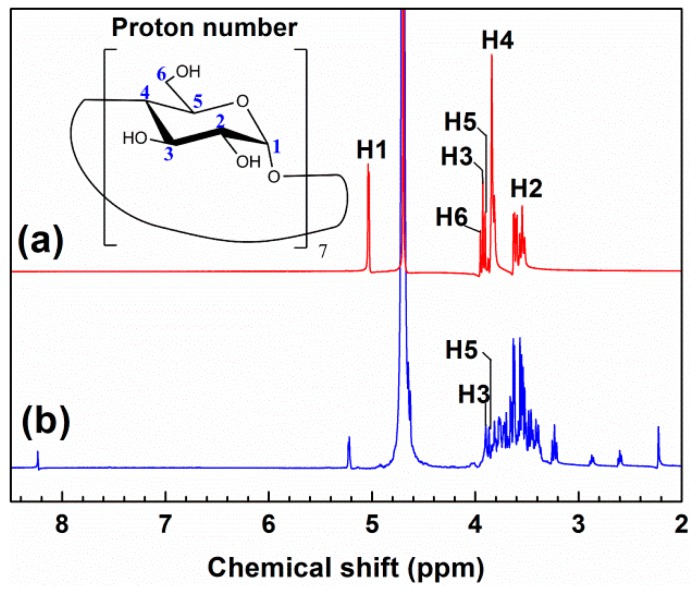
^1^H NMR spectra of (**a**) β-cyclodextrin and (**b**) soluble cyclodextrin polymer in D_2_O.

The prepared SCDP as the host is the basis of accommodating the guest moiety (CA) to construct the stable molecular assembly; hence, it is of value to characterize and confirm the structure of SCDP. The crosslinking structure of SCDP can be directly verified by the NMR technique. Figure 1 shows the ^1^H NMR spectra of β-CD and SCDP. It is worth mentioning for the proton number (inset) that H3 and H5 locate on the interior of the β-CD cavity, while H2 and H4 locate outside the cavity. Furthermore, H1 locates near the wide rim, while H6 is on the opposite side near the narrow rim. As shown in Figure 1, negligible variation in chemical shift was observed for H3 (3.927 ppm) and H5 (3.904 ppm), which indicates that little change occurs in the chemical environment of the inner cavity of β-CD after the crosslinking reaction. The unchanged property of the hydrophobic cavity of SCDP is conducive to include the resembled guest moiety with the inclusion process of β-CD. The occurrence of the crosslinking reaction can be evidenced by comparing the spectra before and after crosslinking because both upfield and downfield shifts are visible for other protons (H1, H2, H4 and H6) on the β-CD subunit. This means that almost all the external hydroxyls of the β-CD subunit are active sites for crosslinking with glutaraldehyde molecules. In addition, the reduced intensity of protons on the outer surface of β-CD after crosslinking also demonstrates the eventual formation of polymerized product [27].

**Figure 2:**
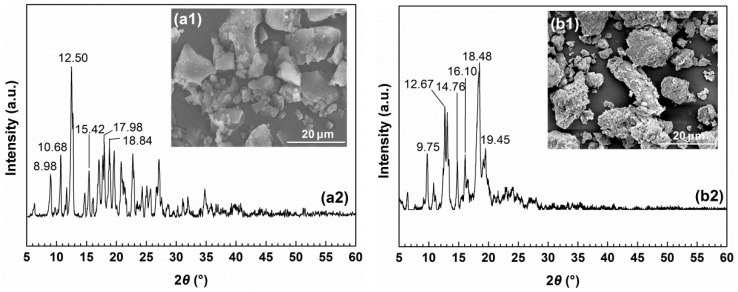
Microstructures of (**a1**) β-cyclodextrin (β-CD) and (**b1**) soluble cyclodextrin crosslinking polymer (SCDP) along with the corresponding XRD patterns of (**a2**) β-CD and (**b2**) SCDP.

The morphologies and XRD patterns for β-CD before and after crosslinking are presented in Figure 2. It is clear in Figure 2a1 that β-CD exhibits the regular block form, which is in line with the previous observation in the related studies [22,28]. A series of sharp peaks shown in Figure 2a2, especially at 8.98°, 10.68°, 12.50°, 15.42°, 17.98° and 18.84° of 2*θ*, indicate the crystalline nature of β-CD [29]. In reference to SCDP, irregular aggregation can be observed in Figure 2b1; meanwhile, sharp peaks at 9.75°, 12.67°, 14.76°, 16.10°, 18.48° and 19.45° (2*θ*) in Figure 2b2 also reveal the crystalline property of SCDP. The crystallization behavior of the as-prepared SCDP differs prominently from that of insoluble polymerized species in resin or gel form. Most of the insoluble cyclodextrin polymers are amorphous owing to the random stacking of crosslinking chains [6,28]. In this study, β-CD subunits are crosslinked by flexible glutaraldehyde based on the weak interactions between carbonyl and hydroxyl groups, which may not have a significant impact on the inherent crystalline property of β-CD. Closer inspection of Figure 2 indicates that the intensity and half-peak width of the main diffraction peaks for SCDP are different from that of β-CD. This implies that the crystallinity and the crystallite dimension are changed after crosslinking owing to the introduction of glutaraldehyde. 

According to the analyses of the ^1^H NMR and XRD results, it is reasonable to state that the including ability of the β-CD subunit may be unchanged after crosslinking. Moreover, the weak crosslinking polymer still keeps the crystalline property to a certain extent.

### 3.2. Characterization of Molecular Assembly

**Figure 3:**
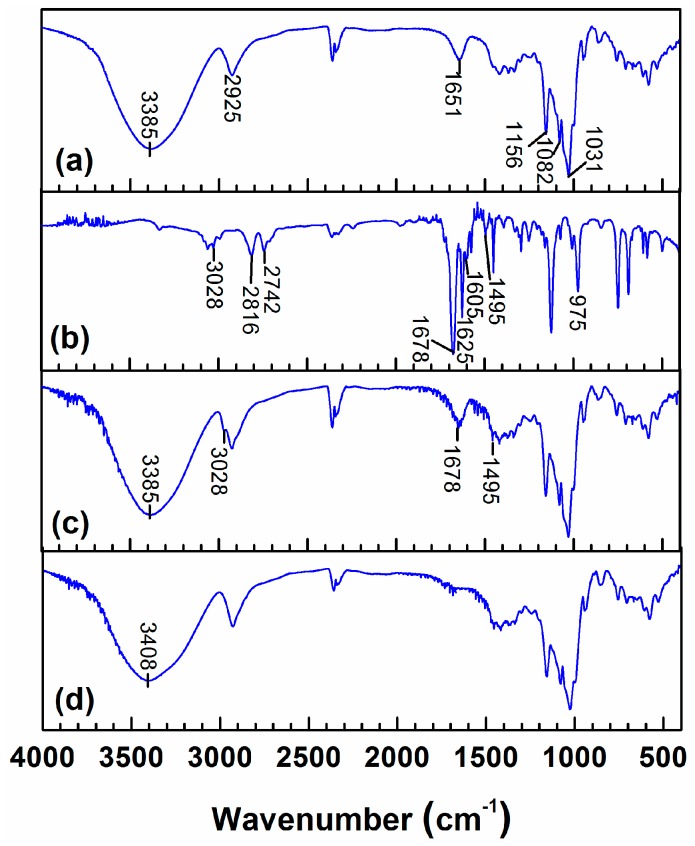
FTIR spectra of (**a**) soluble cyclodextrin polymer, (**b**) *trans*-cinnamaldehyde, (**c**) their physical mixture, and (**d**) SCDP/CA assembly.

In light of the reserved including ability of crosslinking host structure, the molecular assembly between SCDP and CA was prepared and characterized. Considering the existence of a carbonyl group in the guest moiety (CA), FTIR analyses could be conducted to demonstrate the formation of assembly [30], the spectra of which are depicted in Figure 3. In Figure 3a, the spectrum of β-CD shows the characteristic adsorption coinciding with the previous reports [22,31]; the broad peak at 3385 cm^−1^ is attributed to the stretching vibration of –OH; the peak at 2925 cm^−1^ stems from the symmetric stretching vibration of –CH_2_–; the peaks at 1082 and 1031 cm^−1^ can be assigned to the symmetric and asymmetric stretching vibration of C–OH; the adsorption band between 1422 and 1333 cm^−1^ is the characteristic of in-plane deformation vibration for –OH; the peak at 1156 cm^−1^ can be attributed to the asymmetric stretching vibration of C–O–C. For the spectrum of CA in Figure 3b, the peak at 3028 cm^-1^ is credited to the stretching vibration of –CH– (benzene) with its out-of-plane deformation vibration locating at the adsorption band below 900 cm^−1^; two adsorption bands at 2816 and 2742 cm^−1^ are ascribed to the stretching vibration of OC–H; the peak at 1678 cm^−1^ is ascribed to the stretching vibration of C=O; the peak at 1625 cm^−1^ is assigned to the stretching vibration of –C=C– with the corresponding deformation vibration at 975 cm^−1^; and the weak adsorption band in the range from 1605 to 1495 cm^−1^ can be credited to skeleton vibration of the benzene ring. Figure 3c provides the spectrum of the physical mixture of SCDP and CA, which seems to be the simple superposition of the individual spectra in the respective Figure 3a,b. For instance, the characteristic peak (3385 cm^−1^) belonging to β-CD is found in Figure 3c; whilst the characteristic peaks of CA (3028, 1678 and 1495 cm^−1^) are also observed in this spectrum. The spectrum of SCDP/CA assembly denoted in Figure 3d is akin to that of β-CD due to its higher molecular weight compared with CA [32,33]. It is worth further attention that the peak (1651 cm^−1^, Figure 3a) assigned to bound water in the interior of the β-CD cavity vanishes in the spectrum of SCDP/CA assembly. This is direct evidence for the inclusion of CA in the hydrophobic cavity of SCDP. Moreover, the vibration of –OH exhibits a blueshift from 3385 to 3408 cm^−1^, which may be caused by the formed hydrogen bond between SCDP and CA [21,22]. The variation of shift and intensity of FTIR absorption peaks for host or guest moiety can also confirm the formation of molecular assembly [34]. Based on FTIR spectra, few covalent bonds are probably formed during the assembling process, and thus only non-covalent interactions occur between host and guest moieties.

The apparent stability constant (*K*_s_, mol^−1^) of the resulting assembly can be qualitatively evaluated by phase solubility studies on the simple β-CD/CA complex through the equation as follows:

(1) $$K_{s}=\frac{\mathrm{slope}}{S_{0}(1-\mathrm{slope})}$$

where *S*_0_ is the inherent solubility of CA in water, mol/L; and slope means the slope of the phase solubility curve. In addition, the assembling thermodynamics parameters can be derived from *K*_s_ at different temperatures by the integrated form of the van’t Hoff equation and classical relationship:

(2) $$\ln K_{s}=-\frac{\Delta H_{a}}{RT}+\frac{\Delta S_{a}}{R}$$

(3) $$\Delta G_{a}=\Delta H_{a}-T\Delta S_{a}$$

where *R* is the universal gas constant, J/(mol·K); *T* is the temperature, K; Δ*G*_a_ (J/mol), Δ*H*_a_ (J/mol) and Δ*S*_a_ (J/(mol·K)) denote the Gibbs free energy, enthalpy and entropy changes of the assembling process, respectively. The phase solubility curves of the β-CD/CA complex at pre-specified temperatures are shown in Figure S1, and the corresponding parameters are listed in Table S2. 

From Figure S1, all the phase solubility curves display an A_L_ type at the studied temperatures that means a linear increase of CA solubility in water with the rise of β-CD concentration [25,35]. This type of phase solubility curve indicates a 1:1 stoichiometry ratio of host:guest in the complex [25]. It can be found in Table S2 that the solubility of CA in water increases as the temperature increases. However, the value of *K*_s_ decreases with the elevated temperature suggesting an exothermic inclusion process between CA and β-CD, which is also demonstrated by the negative sign of Δ*H*_a_. The negative value of Δ*S*_a_ indicates that the inclusion process is prone to make the system more orderly. Furthermore, the negative value of Δ*G*_a_ reveals a spontaneous inclusion process between β-CD and CA. The similar thermodynamic characteristics for the β-CD/CA complex were observed by Hill and co-workers [24].

**Figure 4:**
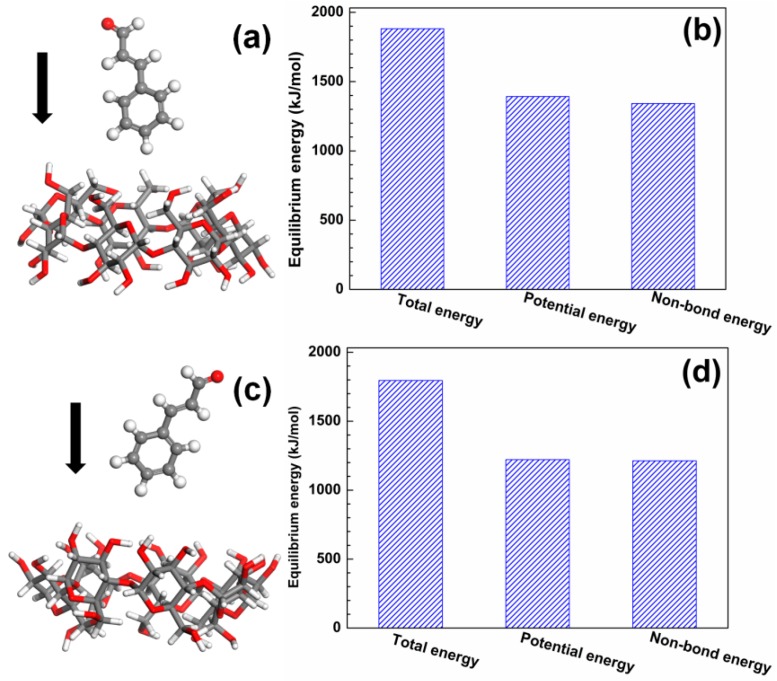
Molecular dynamics simulations for the β-CD/CA complex at 303 K: (**a**, **b**) equilibrium energies for CA assembled through the narrow rim of β-CD, and (**c**, **d**) equilibrium energies for CA assembled through the wide rim of β-CD.

The explicit configuration information of the β-CD/CA complex favors clarification of the distribution of CA in the network of SCDP. Therefore, it is necessary to simulate the inclusion process between β-CD and CA via the molecular dynamics method and acquire the corresponding energies. Owing to the structural property of β-CD, the CA molecule can be encapsulated through either the narrow or the wide rim of the host molecule. Thus, two possible configurations of the β-CD/CA complex could exist, whose simulation results are presented in Figure 4, along with the fluctuations of temperature and various energies given in Figure S2. In Figure S2, the temperature stayed constant at 303 K in the simulating period, and all the energies, namely total energy (*E*_t_), potential energy (*E*_p_) and non-bond energy (*E*_n_), tended to be stable at the end of the simulation. In respect to the assembling orientation shown in Figure 4a, the equilibrium values of *E*_t_, *E*_p_ and *E*_n_ for the complex are 1881.7, 1392.9 and 1342.1 kJ/mol, respectively. For the assembling orientation shown in Figure 4c, the equilibrium values of *E*_t_, *E*_p_ and *E*_n_ for the complex are 1795.4, 1221.3 and 1213.7 kJ/mol, respectively. The values of *E*_t_, *E*_p_ and *E*_n_ for the complex assembled via the narrow rim of β-CD are higher than those of the complex assembled via the wide rim. The steric effect of narrow rim assembling is more pronounced than that of wide rim assembly, which is conducive to forming intensive non-covalent interactions (e.g., van der Waals’ force, hydrogen bond and hydrophobic effect) between host and guest moieties. The enhanced non-covalent interactions result in elevated *E*_n_ and *E*_p_, and thus increase the value of *E*_t_ [36].

A further comparison between the equilibrium energies given in Figure 4b,d reveals that the difference in energy values between two configurations is too indistinctive to distinguish the superior one. Therefore, it is rational to assume that both assembling routes may jointly contribute to the formation of the complex. According to this assumption, CA molecules could be encapsulated in either orientation of the β-CD subunit in the SCDP network without an energy barrier.

### 3.3. Corrosion Inhibition Effect of Molecular Assembly

**Figure 5:**
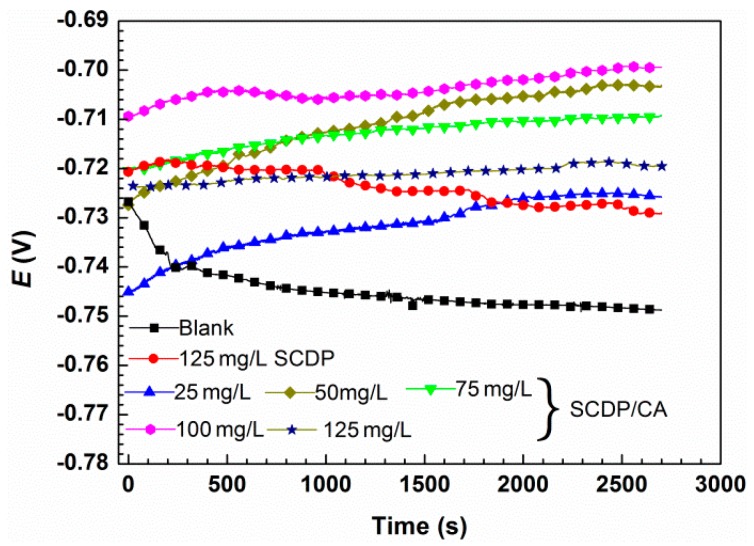
Evolution of *E*_ocp_ with time for mild steel electrodes in 3.5% NaCl solution in the absence and presence of various additives at 30 °C.

Before polarization and EIS determinations, the relationship between *E*_ocp_ and time for mild steel electrodes in 3.5% NaCl solution with and without different additives was recorded at 30 °C. The electrochemical test for the electrode in 3.5% NaCl solution merely containing SCDP was also performed in order to disclose the actual corrosion inhibition mechanism of SCDP/CA assembly. The variation of *E*_ocp_ with time is illustrated in Figure 5. It is obvious from this figure that all the curves display a stable trend with elapsed time. The stable *E*_ocp_ values of electrodes in 3.5% NaCl solution in the presence of additives are nobler than that of the blank control. This result could be assigned to the adsorption of additives on the steel surface [37]. The adsorption of SCDP or SCDP/CA assembly on the steel surface is inclined to shield the electron-rich and negatively charged region on the metal surface [38]. As a result, the electrode potential is promoted in the presence of additives. Moreover, *E*_ocp_ monotonously increases with the increasing concentration of SCDP/CA assembly indicating an enhanced surface adsorption and corrosion inhibition effect.

**Figure 6:**
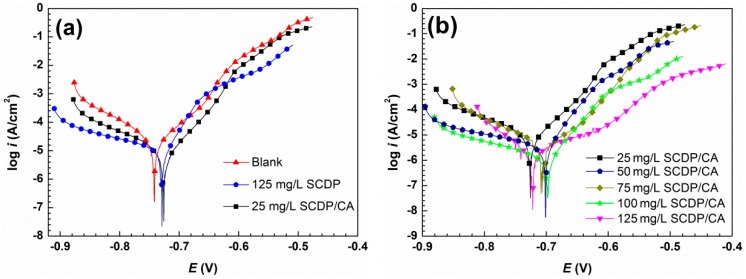
Potentiodynamic polarization curves for mild steel electrodes in 3.5% NaCl solution at 30 °C: (**a**) blank, in the presence of 125 mg/L SCDP and 25 mg/L SCDP/CA assembly; (**b**) in the presence of different concentrations of SCDP/CA assembly.

In order to obtain the corrosion kinetic characteristics, potentiodynamic polarization measurements were conducted under stable *E*_ocp_ at 30 °C for mild steel electrodes in 3.5% NaCl solution with and without various additives, the results of which are shown in Figure 6. It is evident from Figure 6a that the polarization curves moved to the region of lower current density and both cathodic and anodic branches were simultaneously inhibited in the presence of 125 mg/L SCDP or 25 mg/L SCDP/CA assembly. This result is indicative of the adsorption of additives on the steel surface. With respect to mild steel immersion in 3.5% NaCl solution, reduction of oxygen and dissolution of iron constitute cathodic and anodic electrochemical reactions, respectively [39]. Special inspection of Figure 6a indicates that the inhibition of the anodic branch in the presence of 25 mg/L SCDP/CA assembly is more significant than that in the presence of 125 mg/L SCDP suggesting an enhanced inhibition effect of anodic dissolution by the molecular assembly. Figure 6b denotes the polarization curves for steel electrodes in 3.5% NaCl solution with different concentrations of SCDP/CA assembly. It is seen that the cathodic and anodic branches are further suppressed as the concentration of assembly increases. This suggests the corrosion inhibition efficiency can be promoted with the increasing concentration of SCDP/CA assembly. 

The corresponding kinetic parameters, namely corrosion potential (*E*_corr_), corrosion current density (*i*_corr_), cathodic (*b*_c_) and anodic (*b*_a_) Tafel slopes were obtained by Tafel extrapolation and are tabulated in Table 1. The corrosion inhibition efficiency (IE_1_, %) derived from *i*_corr_ was calculated by the following equation:

(4) $${\mathrm{IE}}_{1}=\frac{i_{\mathrm{corr}}^{0}-i_{\mathrm{corr}}^{i}}{i_{\mathrm{corr}}^{0}}\times 100\%$$

where $i_{\mathrm{corr}}^{0}$ and $i_{\mathrm{corr}}^{i}$ are the corrosion current densities for electrodes in 3.5% NaCl solution without and with additives, mA/cm^2^, respectively. It is obvious from Table 1 that SCDP/CA assembly is more efficient than SCDP in retarding the corrosion of mild steel in 3.5% NaCl solution for lower *i*_corr_. The value of IE_1_ for the electrode in the solution with 125 mg/L SCDP is only 44.5%, which is lower than that with 25 mg/L SCDP/CA assembly (80.6%). The electronegative oxygen atoms in the hydroxyl groups of the SCDP skeleton can be attracted by the oxidative metal surface with a positive charge [40]. In addition, the high molecular weight of SCDP also facilitates its physical coverage on the surface of mild steel. The stack layer of SCDP may prevent the steel surface from ions and dissolved oxygen in the bulk solution and hence mitigate the corrosion to a certain extent. 

In respect to SCDP/CA assembly, CA as the guest moiety can be dispersed and dissolved in the aqueous solution through host–guest assembly. According to valence bond theory [41], the unoccupied 4s and 4p hybrid orbitals of the iron atom on the steel surface may be correctly oriented towards the oxygen non-bonding electron pairs and the conjugate electrons of the CA molecule. Consequently, the covalent bond might be formed between CA and the iron atom, resulting in the chemisorption of inhibitor on the steel surface. The adsorption of CA would form a hydrophobic layer on the steel surface by virtue of its low molecular polarity, which could isolate the metal from corrosive species in the solution. This also explains the superior corrosion inhibition effect of SCDP/CA assembly as compared to that of SCDP.

Further observation of the data in Table 1 reveals that the value of *i*_corr_ continues to decrease as the concentration of SCDP/CA increases with a high IE_1_ value of 92.2% in the presence of 125 mg/L SCDP/CA assembly. Higher inhibitor concentration will give rise to the higher coverage on the metal surface by inhibitor molecules and therefore decrease the current density of the corrosion reaction [42]. The value of *E*_corr_ has a positive shift in the presence of SCDP/CA assembly, and the maximum variation in *E*_corr_ (44 mV) from that of the uninhibited electrode is less than 85 mV, supporting the characteristics of the mixed type corrosion inhibitor [43]. Furthermore, the change of *b*_a_ is more drastic than that of *b*_c_ after the addition of SCDP/CA assembly, implying a more significant influence on the anodic reaction than that of the cathodic behavior. These results reveal that SCDP/CA assembly acts as the anodic-dominant mixed type corrosion inhibitor.

**Figure 7:**
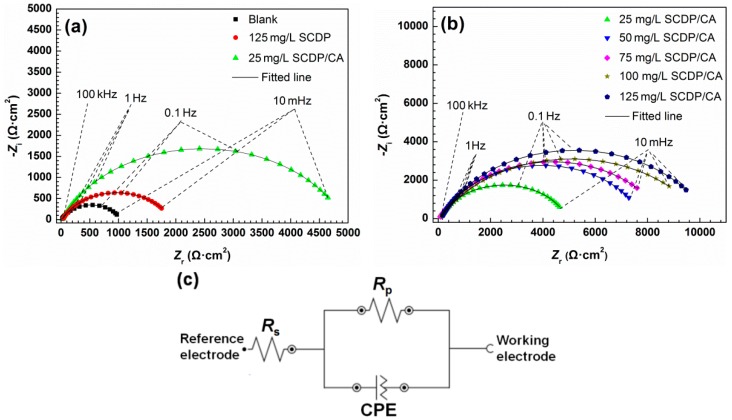
Electrochemical impedance spectra in Nyquist form for mild steel electrodes in 3.5% NaCl solution at 30 °C in the absence and presence of various additives (**a**, **b**), and the corresponding equivalent circuit (**c**).

EIS provides a reliable and nondestructive method to evaluate the inhibitive characteristics of a corrosion inhibitor for metals [44]. Figure 7a,b shows the EIS results in Nyquist form for mild steel electrodes in 3.5% NaCl solution with and without various additives at 30 °C. It is clear in Figure 7a,b that all plots exhibit a single capacitive loop, suggesting the corrosion is controlled by one time constant as the charge transfer process [45]. In addition, each capacitive loop gives the shape of a depressed semicircle with the center below the horizontal axis, which originates from frequency dispersion resulting from the inhomogeneous roughness of the electrode surface. The diameter of the loop shown in Figure 7a is enlarged in the presence of SCDP or SCDP/CA assembly compared with that of the blank control. This signifies that both SCDP and SCDP/CA assembly can improve the corrosion resistance of mild steel in 3.5% NaCl solution. Whereas, the corrosion inhibition effect of SCDP/CA assembly is better than that of SCDP for the larger loop diameter, which is akin to the results of potentiodynamic polarization. In Figure 7b, the diameter of the capacitive loop is further increased with the rise of SCDP/CA assembly concentration indicating the progressively intact layer formed on the steel surface by adsorption of the assembly. 

The Randles equivalent circuit depicted in Figure 7c was utilized to model the experimental data. The constant phase element (CPE) displaces the pure capacitor in order to obtain the accurate fitting results, whose impedance (*Z*_CPE_) can be expressed by the following equation:

(5) $$Z_{\mathrm{CPE}}=\frac{1}{Y_{0}{\left(j\omega \right)}^{n}}$$

where *Y*_0_ is a proportional factor, *j* is an imaginary number and *j*^2^ = −1, *ω* is the angular frequency, and *n* is a deviation parameter (−1 < *n* < 1). The corresponding impedance parameters, such as solution resistance (*R*_s_), polarization resistance (*R*_p_) and double-layer capacitor (*C*_dl_), were fitted and are listed in Table 2. The corrosion inhibition efficiency (IE_2_, %) based on *R*_p_ was calculated by the following relationship:

(6) $${\mathrm{IE}}_{2}=\frac{R_{p}^{i}-R_{p}^{0}}{R_{p}^{i}}\times 100\%$$

where $R_{p}^{0}$ and $R_{p}^{i}$ are the polarization resistances of the electrodes in 3.5% NaCl solution without and with additives, Ω·cm^2^, respectively. 

The parameter *R*_s_ shown in Table 2 displays moderate change with and without different concentrations of assembly indicating the reproducibility of experimental results [37]. The value of *R*_p_ increases from 1006.2 ± 12.9 Ω·cm^2^ (blank) to 10,565.5 ± 72.7 Ω·cm^2^ (125 mg/L SCDP/CA assembly), which can be ascribed to the gradually integrated and uniform adsorbed layer on the steel surface. The values of IE_2_ in Table 2 have the similar variation trend with those calculated from *i*_corr_. The tabulated values of linear polarization resistance (*R*_pl_) for electrodes in 3.5% NaCl solution with and without various additives agree well with the fitted *R*_p_ values, which suggests good stability and linearity of the EIS tests in this study. 

All the values of *n* closing to 1 discloses that CPE behaves mainly as an interfacial capacitor (*C*_dl_). The expression of *C*_dl_ can be described by the Helmholtz model as follows:

(7) $$C_{\mathrm{dl}}=\frac{\varepsilon \cdot \varepsilon^{0}}{d}A$$

where *d* is the thickness of the double-layer, cm; *A* is the effect area of the electrode, cm^2^; *ε*^0^ and *ε* are the dielectric constants of vacuum and adsorbed layer, F/cm, respectively. The decrease in *C*_dl_ values from 782.3 μF/cm^2^ (blank) to 180.7 μF/cm^2^ (125 mg/L SCDP/CA assembly) is related to the distribution of charge at the metal/solution interface. The adsorption of organic substance displaces the originally adsorbed water molecules and ions along the outer Helmholtz plane resulting in a decrease of dielectric constant and increase of double-layer thickness. According to Equation (7), the value of *C*_dl_ monotonously decreases with increasing concentration of SCDP/CA assembly. 

Based on all electrochemical results, a conclusion can be drawn that SCDP/CA assembly can mitigate the corrosion of mild steel in a 3.5% NaCl solution, which is more efficient than SCDP.

### 3.4. Surface Analyses

**Figure 8:**
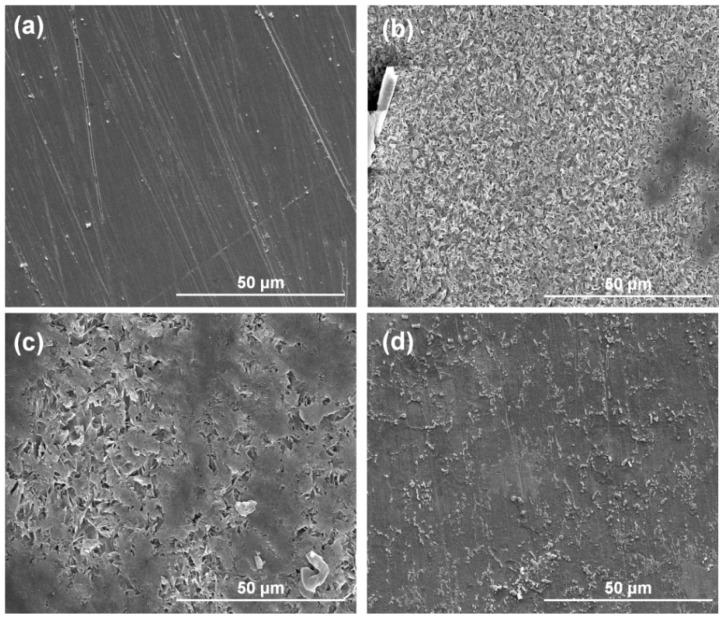
Surface morphologies of the steel specimens after 72 h immersion in 3.5% NaCl solution at 30 °C: (**a**) freshly polished specimen, (**b**) in the absence of any additive, (**c**) in the presence of 125 mg/L SCDP, and (**d**) 125 mg/L SCDP/CA assembly.

The SEM images of steel specimens after 72 h immersion in 3.5% NaCl solution at 30 °C with and without different additives are shown in Figure 8, along with the freshly polished specimen for comparison. As shown in Figure 8a, apparent scratches caused by mechanical polishing can be clearly observed on the steel surface. On the contrary, the appearance of the specimen without any additive shown in Figure 8b is seriously damaged with numerous cracks and holes. This outcome reveals that the attack of corrosive medium leads to substantial corrosion of the metallic surface without inhibitor. In Figure 8c, the morphology of the specimen inhibited by 125 mg/L SCDP is protected to a certain degree; however, the distinct defects can be also seen on the surface indicating the limited corrosion inhibition effect of SCDP. The specimen inhibited by 125 mg/L SCDP/CA assembly exhibits a drastic reduction in surface roughness in Figure 8d, which is ascribed to the adsorption of inhibitor on the steel surface. SEM examinations strongly support the conclusion drawn in the electrochemical analyses that SCDP/CA assembly can effectively inhibit the corrosion of mild steel in 3.5% NaCl solution.

**Figure 9:**
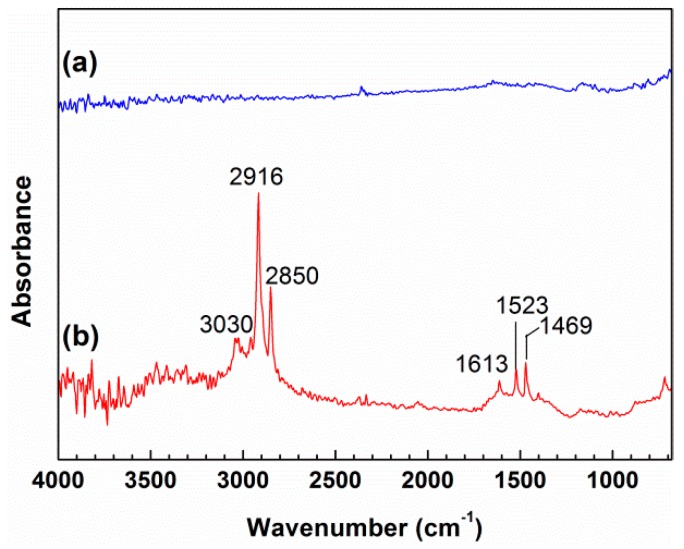
ATR-FTIR spectra of (**a**) freshly polished steel specimen, and (**b**) steel specimen after 72 h immersion in 3.5% NaCl solution at 30 °C with 125 mg/L SCDP/CA assembly.

It has been explicit from the aforementioned analyses that the efficient corrosion inhibition of SCDP/CA assembly stems from its strong adsorption on the steel surface. Therefore, it is essential to clarify the specified adsorption behavior of SCDP/CA assembly. The ATR-FTIR spectra of the freshly polished steel specimen and the specimen after 72 h immersion in 3.5% NaCl solution at 30 °C with 125 mg/L SCDP/CA assembly are presented in Figure 9. The spectrum of the freshly polished specimen lacks the adsorption band due to the inherent characteristics of metals. However, there are a series of characteristic peaks in the spectrum of the inhibited specimen by 125 mg/L SCDP/CA assembly. The peak at 3030 cm^−1^ should be attributed to the stretching vibration of –CH– (benzene); the peaks at 2916 and 2850 cm^−1^ can be assigned to the stretching vibration of C–H on the aldehyde group; the peak at 1613 cm^−1^ arises from the stretching vibration of C=O; and the peaks at 1523 and 1469 cm^−1^ can be ascribed to the stretching vibration of C=C [24]. It is noteworthy that little characteristic sign of the host moiety (SCDP) was detected in the spectrum of the inhibited specimen, such as a hydroxyl group and α-1,4-glycosidic bond. The shift and intensity change of characteristic peaks for CA are found to be as the standard spectrum illuminating the chemisorption nature of the inhibitor [24], which is in accordance with the polarization analyses in Section 3.3. Hence, the assumption can be made on the adsorption mechanism of SCDP/CA assembly that the guest molecule (CA) can be released from SCDP and solely adsorbed on the steel surface, and as a result a hydrophobic layer is formed to isolate the metal from the corrosive species. The strength of the chemical interaction between CA and the iron atom is stronger than the non-covalent bond existing in the assembly, which may be the driving force for CA to be released from the cavity of SCDP. 

**Figure 10:**
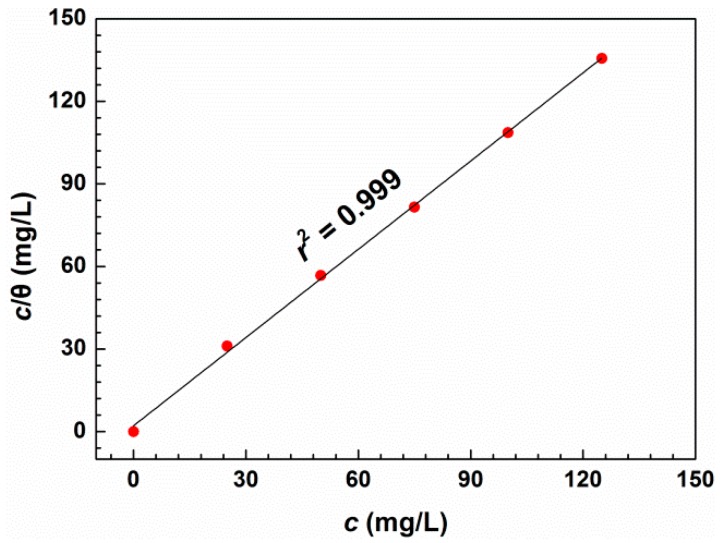
Langmuir model for the adsorption of SCDP/CA assembly on mild steel in 3.5% NaCl solution at 30 °C.

Adsorption isotherms are frequently used to describe the distribution of adsorbed species on the metal/solution interface [46]. Among most typical models, the Langmuir adsorption isotherm, based on the coverage data (*θ*) from polarization tests, achieved a satisfactory level of fitting with a correlation coefficient of 0.999 (Figure 10), which can be represented by the following formulae:

(8) $$\theta =\frac{{\mathrm{IE}}_{1}}{100}$$

(9) $$\left(\frac{\theta }{1-\theta }\right)=K_{\mathrm{ads}}c$$

where *c* is the concentration of SCDP/CA assembly, mg/L; and *K*_ads_ is the adsorption equilibrium constant. The straight fitted line with the slope close to unity indicates the adsorption of guest molecules on the steel surface is a monolayer in nature [47].

### 3.5. Theoretical Studies

**Figure 11:**
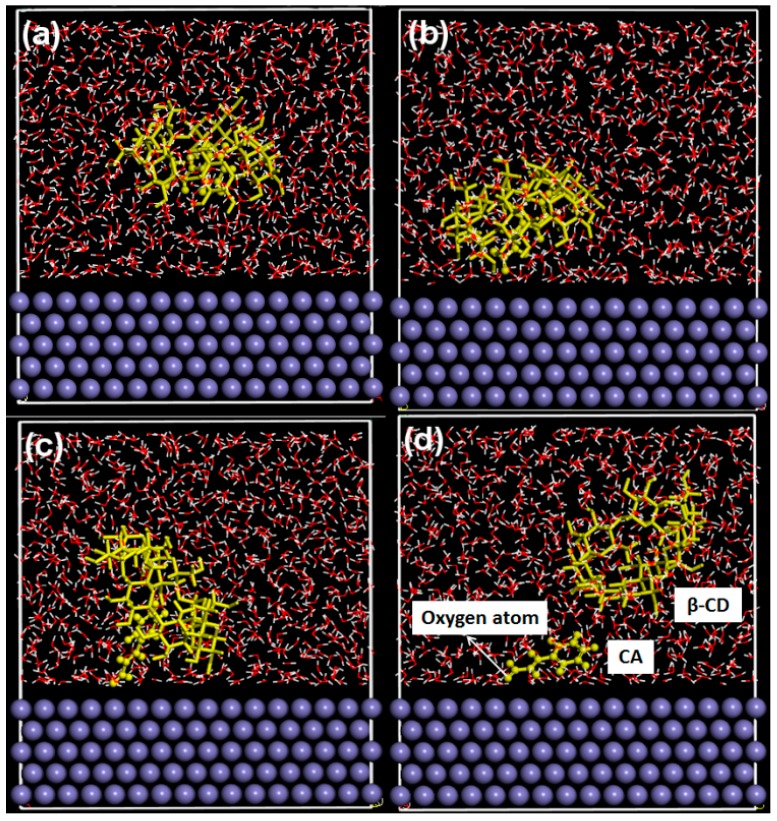
Molecular dynamics simulations for the adsorption process of the β-CD/CA complex on the surface of mild steel at 303 K: (**a**) 0 ps, (**b**) 90 ps, (**c**) 170 ps, (**d**) 230 ps.

Molecular dynamics simulation is a powerful tool to clarify the adsorption mechanism of organic molecules on the metal surface [40,45,48]. Allowing for the calculation cost and complexity, one representative crosslinking unit (i.e., β-CD/CA complex) was utilized to complete the simulation in this study. The adsorption process for the β-CD/CA complex on a steel surface in an aqueous solution is depicted in Figure 11. Initially, the randomly placed complex is relative stable in water as shown in Figure 11a. The low molecular polarity of the guest moiety (CA) mismatches with that of surrounding environment. Therefore, the CA molecule can be only stabilized in the low polarity cavity of β-CD. Abundant hydroxyl groups on the skeleton of β-CD may be attracted by the iron surface via electrostatic interactions or van der Waals forces [49]; as a consequence, the complex integrally approaches the surface of iron after the 90-ps simulation as shown in Figure 11b. After a simulation period of 170 ps (Figure 11c), the CA molecule shows a propensity to separate itself from the hydrophobic cavity of β-CD with the oxygen heteroatom pointing to the iron surface. In Figure 11d, CA and β-CD molecules are apart from each other thoroughly with the simulation time elapsed: the β-CD molecule diffuses in the bulk water, leaving CA in the adjacent metal surface. The simulated results intuitively support the assumption made by ATR-FTIR analyses.

**Figure 12:**
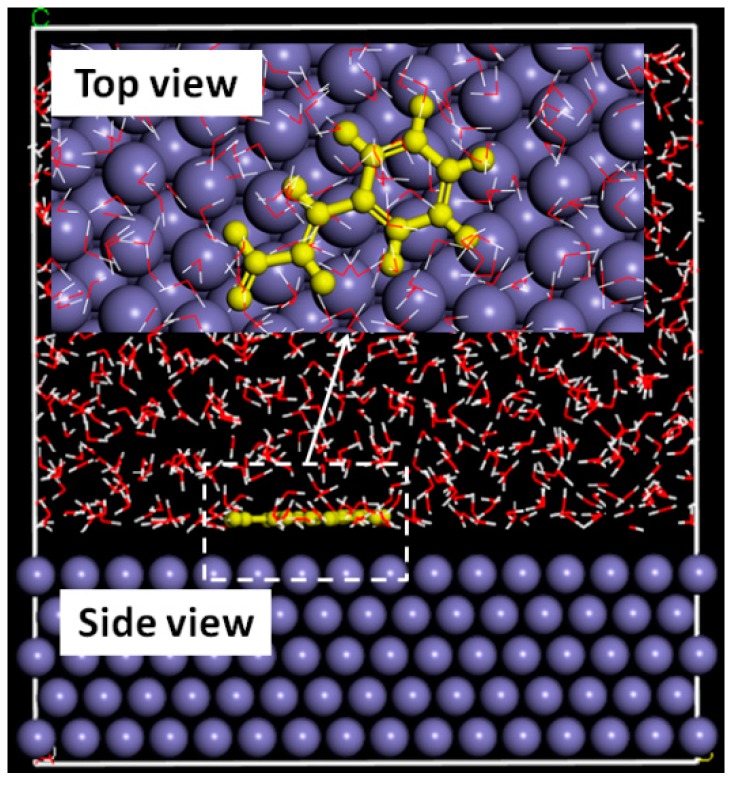
Top and side snapshots of the equilibrium adsorption configuration for the guest molecule (CA) on the iron surface at 303 K (the host moiety is manually removed for clarity).

The equilibrium adsorption configuration of the CA molecule on the iron surface at the end of the simulation is presented in Figure 12. According to the top and side views of the equilibrium configuration, the CA molecule favors adsorbtion on the iron surface in parallel mode. This adsorption configuration is conducive to obtaining large surface coverage accompanied by high corrosion inhibition efficiency [50]. The adsorption (*E*_ads_, kJ/mol) and binding (*E*_bind_, kJ/mol) energies of the β-CD/CA complex on the iron surface are approximated at the equilibrium state of the simulation system, which can be calculated by the following equations:

*E*_ads_ = *E*_total_ − (*E*_iron+water_ + *E*_com+water_) + *E*_water_ (10)

*E*_bind_ = −*E*_ads_ (11)

where *E*_total_ is the energy of the total system, kJ/mol; *E*_iron+water_ is the energy of iron and water, kJ/mol; *E*_com+water_ is the energy of the β-CD/CA complex and water, kJ/mol; and *E*_water_ is the energy of entire water molecules, kJ/mol. The calculated values of *E*_ads_ and *E*_bind_ are −469.2 and 469.2 kJ/mol, respectively. The negative sign of *E*_ads_ indicates the spontaneous process for the complex adsorbed on the metal surface with high binding strength [51].

Regarding the mechanism of guest release from cyclodextrins, Lis et al. [32] raised three models: activation, diffusion and polymeric breakdown. However, taking both experimental and theoretical results of this study into consideration, the classical mechanisms of guest release may not be applicable for SCDP/CA assembly on the steel surface in aqueous solution. Herein, an energy competition principle might be appropriate to explain the adsorption behavior of the assembly on the metal surface. The guest component would get rid of the weak non-covalent binding and release from the cavity of cyclodextrins when the strong interaction with high binding energy is formed between the guest molecule and metal atom. 

In order to reveal the parallel adsorption mechanism of the CA molecule on the steel surface, quantum chemistry parameters (i.e., optimal configuration, MEP, HOMO and LUMO distributions) were calculated and are illustrated in Figure S3. According to frontier orbital theory [52], the active sites of organic molecules mainly occurred on HOMO and LUMO, which reflect the abilities of electron-donating and -accepting, respectively. A flat configuration is optimal for the CA molecule as shown in Figure S3a. It is clear in Figure S3b that the negative charge is centered at the oxygen atom, while the positive charge distributes over the residual part. The negatively charged region of CA is prone to provide excess electrons to complete the chemisorption on the steel surface. The electronic structure of CA is also featured by distributions of HOMO and LUMO. As shown in Figure S3c, HOMO mainly concentrates on the aldehyde group and the unsaturated bond, which is akin to the distribution of negative electrostatic potential given in Figure S3b. Meanwhile, in Figure S3d, LUMO spreads throughout the entire molecule for the conjugated electronic structure of CA. In addition, this distribution pattern of LUMO is propitious for CA to parallelly adsorb on the steel surface, which can accept the free electrons escaped from the metal surface to the utmost extent and the consequent formation of the back-donating bond [48]. Therefore, the electronic parameters may be responsible for the adsorption behavior of CA on the steel surface: CA is initially attracted by the positively charged steel surface in aqueous solution through the electronegative oxygen atom and is parallelly adsorbed in chemical form. 

## 4. Conclusions

The soluble crosslinking β-CD polymer was prepared based on the weak crosslinking effect of flexible glutaraldehyde. The interior chemical environment of cyclodextrin subunits is unchanged, indicating the reserved inclusion ability of SCDP for guest molecules. The crystallinity and crystallite dimension of SCDP are different from that of β-CD due to the crosslinking reaction. The molecular assembly between SCDP and CA was constructed by the solution method, which might be treated as a spontaneous process with an exothermic property. Guest CA molecules could be assembled through either the wide or the narrow rim of cyclodextrin subunits in SCDP according to the results of energy analyses. 

The SCDP/CA assembly could effectively retard the corrosion of mild steel in 3.5% NaCl solution with a maximum efficiency of 92.2%, which was more efficient than SCDP because of the existence of the guest moiety. In potentiodynamic polarization measurements, SCDP/CA assembly inhibited both the cathodic and anodic reactions of the steel electrode in 3.5% NaCl solution, and the inhibition effect was enhanced as the concentration of assembly increased. Based on EIS results, SCDP/CA assembly could increase the polarization resistance for the electrode in the corrosive medium, which was also promoted by increasing the concentration of assembly. It was observed in surface analyses that the CA molecule as the guest in the assembly could be released from the cavity of cyclodextrin and solely adsorb on the steel surface, which was vividly evidenced by the molecular dynamics simulation. The CA molecule was inclined to adsorb on the metal surface in parallel mode resulting from its electronic characteristics.

The obtained conclusions in this study might provide experience in preparing the soluble polymers based on cyclodextrins and developing their functional assembly.

## Figures and Tables

**Table 1:** Electrochemical kinetic parameters for mild steel electrodes in 3.5% NaCl solution in the absence and presence of various additives at 30 °C.

Concentration	*E*_corr_ (V)	*i*_corr_ (×10^−1^ mA/cm^2^)	*b*_a_ (mV/dec)	*b*_c_ (mV/dec)	IE_1_ (%)
Blank	−0.742	3.93	56.5	−85.8	—
125 mg/L SCDP	−0.728	2.18	98.7	−83.6	44.5
25 mg/L SCDP/CA	−0.726	0.761	83.4	−98.9	80.6
50 mg/L SCDP/CA	−0.701	0.468	106.9	−81.2	88.1
75 mg/L SCDP/CA	−0.706	0.313	164.4	−89.2	92.0
100 mg/L SCDP/CA	−0.698	0.311	171.3	−87.1	92.1
125 mg/L SCDP/CA	−0.721	0.307	186.6	−106.4	92.2

**Table 2:** Impedance parameters for mild steel electrodes in 3.5% NaCl solution at 30 °C in the absence and presence of various additives.

Concentration	*R*_s_ (Ω·cm^2^)	*C*_dl_ (μF/cm^2^)	*n*	*R*_p_ (Ω·cm^2^)	IE_2_ (%)	*R*_pl_ (Ω·cm^2^)
Blank	11.5	782.3	0.89	1006.2 ±12.9	—	1018.7
125 mg/L SCDP	21.7	511.2	0.91	1859.9 ± 37.7	45.9	1798.9
25 mg/L SCDP/CA	28.3	348.8	0.93	4814.3 ± 61.1	79.1	4827.3
50 mg/L SCDP/CA	33.6	226.4	0.89	7565.4 ± 50.9	86.7	7701.5
75 mg/L SCDP/CA	50.1	258.9	0.87	8385.2 ± 94.4	88.0	8381.4
100 mg/L SCDP/CA	30.9	185.1	0.89	9891.6 ± 68.3	89.8	9780.2
125 mg/L SCDP/CA	55.8	180.7	0.93	10565.5 ± 72.7	90.5	10011.7

## Supplementary Materials

The following are available online at https://www.mdpi.com/2073-4360/11/4/635/s1, Figure S1: Phase solubility curves of *trans*-cinnamaldehyde with β-cyclodextrin at 303, 313 and 318 K; Figure S2: Fluctuation of temperature (a) and energies for *trans*-cinnamaldehyde assembled with β-cyclodextrin through the narrow rim (b) and wide rim during molecular dynamics simulation.; Figure S3: Quantum chemistry descriptors of guest molecule (*trans*-cinnamaldehyde): (a) optimal configuration, (b) mapping of molecular electrostatic potential, (c) HOMO distribution, and (d) LUMO distribution. Table S1: Main composition of Q235A mild steel obtained from optical emission spectroscopy; Table S2: Apparent stability constants and thermodynamic parameters of β-cyclodextrin/*trans*-cinnamaldehyde inclusion complex.
