# Supplementary material for: Molecular Assembly between Weak Crosslinking Cyclodextrin Polymer and trans-Cinnamaldehyde for Corrosion Inhibition towards Mild Steel in 3.5% NaCl Solution: Experimental and Theoretical Studies
Source: Polymers (Basel). 2019 Apr 8;11(4):635. doi: 10.3390/polym11040635 (PMC6523557; doi:10.3390/polym11040635)
Supplement: Supplementary file 1 [file polymers-11-00635-s001.pdf]

Supplementary materials

Molecular Assembly between Weak Crosslinking Cyclodextrin Polymer and *trans*-Cinnamaldehyde for Corrosion Inhibition towards Mild Steel in 3.5% NaCl Solution: Experimental and Theoretical Studies

Yucong Ma [1], Baomin Fan [1,*], Tingting Zhou [1], Hua Hao [2], Biao Yang [1,*] and Hui Sun [1]

[1] School of Materials and Mechanical Engineering, Beijing Technology and Business University, Beijing 100048, China; jzfbm@163.com

[2] Institute of Chemistry, Chinese Academy of Sciences, Beijing 100190, China; haohua@iccas.ac.cn

* Correspondence: fanbaomin@btbu.edu.cn (B. F.); ybiao@th.btbu.edu.cn (B.Y.) Tel.: +86-1358-157-2938 (B.F.)

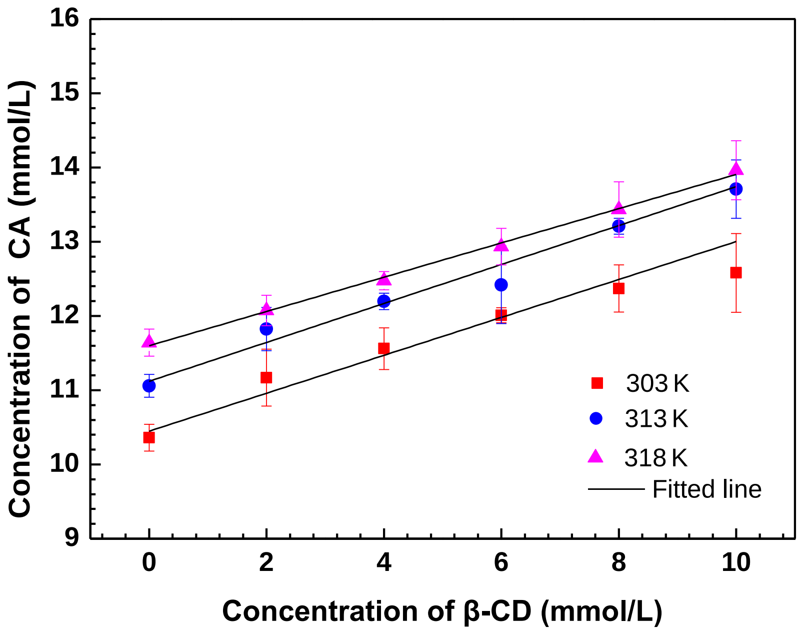

Figure S1. Phase solubility curves of *trans*-cinnamaldehyde with β-cyclodextrin at 303, 313 and 318 K.

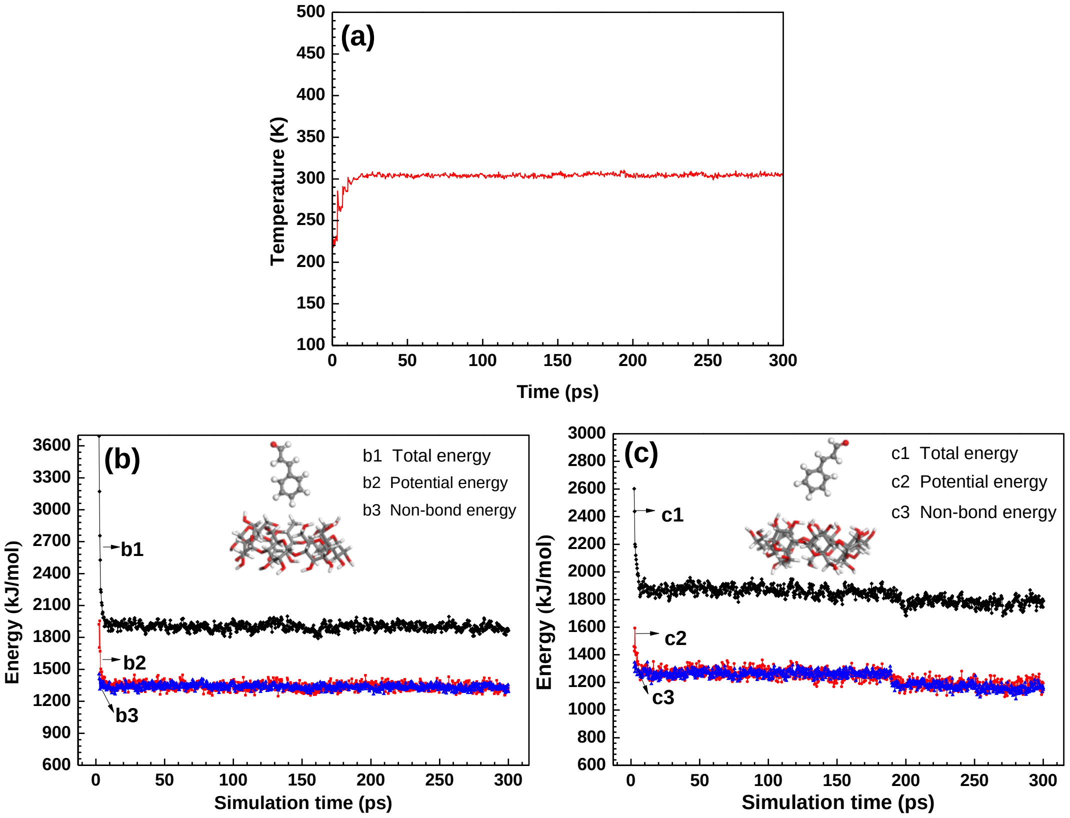

Figure S2. Fluctuations of (a) temperature and (b) energies for *trans*-cinnamaldehyde assembled with β-cyclodextrin through the narrow rim and wide rim during molecular dynamics simulation.

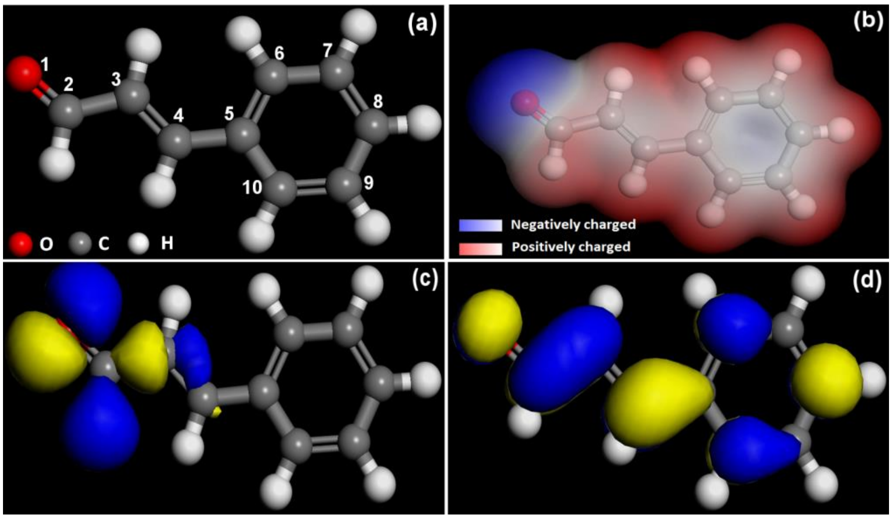

Figure S3. Quantum chemistry descriptors of guest molecule (*trans*-cinnamaldehyde): (a) optimal configuration, (b) mapping of molecular electrostatic potential, (c) HOMO and (d) LUMO distributions.

Table S1. Main composition of Q235A mild steel obtained from optical emission spectroscopy.

Element	C	Mn	Si	S	P	Fe
Content (wt%)	0.16	0.5	0.3	0.05	0.05	balance

Table S2. Apparent stability constants and thermodynamic parameters of β-cyclodextrin/*trans*-cinnamaldehyde inclusion complex.

Temperature (K)	K_s (mol^{-1})	ΔH_a (J/mol)	ΔS_a (J/(mol·K))	ΔG_a (J/mol)
303	34.3			8954.8
313	31.1	-14054.2	-16.8	—
318	25.8			—
